# The natural polyphenol curcumin induces apoptosis by suppressing STAT3 signaling in esophageal squamous cell carcinoma

**DOI:** 10.1186/s13046-018-0959-0

**Published:** 2018-12-05

**Authors:** Ying Liu, Xinhua Wang, Shuang Zeng, Xiane Zhang, Jimin Zhao, Xiaoyan Zhang, Xinhuan Chen, Wanjing Yang, Yili Yang, Ziming Dong, Jingyu Zhu, Xin Xu, Fang Tian

**Affiliations:** 1 Department of Pathophysiology, School of Basic Medical Sciences, Zhengzhou University, Zhengzhou, Henan 450001 People’s Republic of China; 2 Henan Provincial Cooperative Innovation Center for Cancer Chemoprevention, Zhengzhou, Henan 450001 People’s Republic of China; 3 Clinical Research Center, People’s Hospital of Zhengzhou, Zhengzhou, Henan 450001 People’s Republic of China; 4 Department of Histology and Embryology, School of Basic Medical Sciences, Zhengzhou University, Zhengzhou, Henan 450001 People’s Republic of China; 5 Suzhou Institute of Systems Medicine, Center for Systems Medicine, Chinese Academy of Medical Sciences, Suzhou, Jiangsu 215123 People’s Republic of China; 6 School of Medicine and Pharmaceutics, Jiangnan University, Wuxi, Jiangsu 214000 People’s Republic of China

**Keywords:** Curcumin, STAT3, Esophageal squamous cell carcinoma, Apoptosis, PDX model

## Abstract

**Background:**

We and others have previously shown that the STAT3 signaling pathway is activated in some esophageal squamous cell carcinoma (ESCC) cells and is required for the survival and growth of these primary ESCC-derived xenografts. It has also been shown that the natural polyphenol curcumin is an effective anti-tumor agent.

**Methods:**

Luciferase assay and immunoblotting were performed to examine whether curcumin suppressed STAT3 signaling. CCK-8 assay and xenografts were utilized for analyzing ESCC cell growth in culture and mice. Soft agar assay was carried out to determine the colony formation ability of ESCC cells in the presence or absence of curcumin. Cell death and cell cycle were assessed by In CELL Analyzer 2000. Immunohistochemistry and TUNEL assay were used for detecting apoptosis in ESCC tisuses. Molecular docking was performed to evaluate the interaction of curcumin with JAK2. JAK2 activity was assessed using an in vitro cell-free system. HE staining was used to evaluate the ESCC tissues.

**Results:**

The natural polyphenol curcumin inhibited STAT3 phosphorylation rapidly and blocked STAT3-mediated signaling in ESCC cells. It also induced growth arrest and apoptosis in cultured ESCC cells, which were attenuated by enforced expression of STAT3. Furthermore, curcumin preferentially blocked the growth of primary ESCC-derived xenografts that harbored activated STAT3.

**Conclusions:**

Curcumin is able to exert anti-tumor action through inhibiting the STAT3 signaling pathway. Giving its wide use in traditional medicines with low toxicity and few adverse reactions, it is conceivable that curcumin might be further explored as a unique STAT3 inhibitor for anti-cancer therapies.

## Introduction

Esophageal cancer is the eighth most common malignancy and the sixth most common cause of death from malignancy worldwide [1]. Esophageal squamous cell carcinoma (ESCC) is the most common type of esophageal cancers and accounts for 90% of the cases, whose 5-year survival rate remains less than 30% [2]. The development of ESCC is a multifactorial, multistage and complex biological process involving multiple gene abnormalities [2]. As early as 1850s, Virchow found that inflammation was associated with cancers [3]. Recent studies demonstrated that inflammation could initiate and promote malignant transformation, whereas the genetic and epigenetic changes of malignant cells could generate the inflammatory microenvironment to further support the progression of the tumor [4]. Noteworthily, large number of studies have shown that inflammation-related smoking, alcohol, diet and human papilloma virus (HPV) are all associated closely with the development of ESCC [5].

Curcumin is the main natural polyphenol found in the rhizome of *Curcuma longa* (turmeric). Many studies showed that curcumin has anti-oxidation, anti-growth, antiarthritic and anti-inflammation actions [6]. In particular, it has been reported that curcumin induces apoptosis, inhibits cell proliferation and migration in human leukemia, colon, prostate, renal and non-small-cell lung cancer [7–9], suggesting that it might be a novel agent for the prevention and treatment of ESCC.

The Janus kinase/signal transducers and activators of transcription (JAK/STAT) pathway plays an essential role in immune response, inflammation, and carcinogenesis [10, 11]. Cytokines bind to the receptors and activates JAKs, which in turn phosphorylates STATs. Dimerized and phosphorylated STATs are then translocated into the nucleus to regulate gene expression. Some of these genes are important in cell proliferation and survival, including cyclins and anti-apoptotic proteins [12]. In particular, STAT3 can be activated in many cells by various cytokines and growth factors, such as IL-6 and EGF family members [13, 14]. Noteworthily, constitutive activation of STAT3 has been found in various human cancers, such as breast cancer, prostate cancer, ovarian cancer, hepatocarcinoma, and it has shown that activation of STAT3 contributes to tumor cell growth, metastasis and angiogenesis [15–18]. Thus, targeting STAT3 is regarded as a promising strategy for developing novel therapeutics.

In this study, we used ESCC cell lines and four ESCC PDX (patient-derived xenograft) models to further explore the activity and mechanism of curcumin. We found that the compound downregulates STAT3 signaling by suppressing JAK2 activation, leading to inhibition of cell growth and clony formation, cell cycle arrest and apoptosis. Furthermore, preventive use of curcumin significantly inhibited tumor growth in ESCC patient-derived xenografts. These results indicated that curcumin is an effective agent for the preventive treatment of ESCC harboring constitutively activated STAT3.

## Materials and methods

### Cells, tissues and chemicals

Esophageal squamous cell carcinoma (ESCC) cell lines EC1, EC9706, KYSE450 and TE13 were provided by Department of Pathophysiology, School of Basic Medicine, Zhengzhou University. All ESCC cell lines were cultured in Dulbecco’s high glucose modified Eagle’s medium (DMEM) supplemented with 10% fetal bovine serum (FBS), 100 μg/ml of penicillin, and 100 units/ml of streptomycin at 37 °C with 5% CO_2_.

The ESCC tumors used for this study were collected from patients enrolled into the First Affiliated Hospital of Zhengzhou University (Zhengzhou, China) with consensus, and approved by the Ethics Committee of Zhengzhou University. None of these patients had received preoperative chemotherapy or preoperative radiation therapy. The fresh tumor specimens were collected at the time of surgical resection and prepared for implantation in immunodeficient mice. All specimens were examined by two pathologists to confirm the malignant tissues. All the tissues were inoculated into the mice within 2 h after the operations.

Curcumin, Z-VAD-FMK and AG490 were purchased from Selleck Chemicals (Houston, TX, USA). Annexin V-FITC Apoptosis Detection Kit was purchased from Beyotime Biotechnology (Shanghai, China).

### Plasmids construction and gene transfection

The human STAT3 cDNA was cloned into pcDNA3.1 vector with a Myc tag as previously described [19, 20]. A STAT3 luciferase construct (STAT3-Luc) was purchased from Beyotime Biotechnology (Shanghai, China). Plasmids were transiently transfected into EC9706 or TE13 cells by Lipofectamine^®^ 2000 (Invitrogen) according to the manufacturer’s instruction.

### Luciferase assay

After transfected with STAT3-Luc or empty vector along with the internal control renilla luciferase by Lipofectamine^®^ 2000 (Invitrogen) for 24 h, TE13 cells were incubated with indicated agents for 12 h, and then stimulated with 50 ng/ml IL-6 (Novoprotein, Shanghai, China) or vehicle control for 20 min. Cells were then prepared for luciferase assay by using Dual-Luciferase^®^ Reporter Assay System (Promega, Madison, WI, USA) as described previously [21, 22].

### Immunoblotting analysis

Whole cell lysates were prepared for immunoblotting as described previously [23–25]. Equal amounts of total proteins (30 μg) were subjected to SDS-PAGE, transferred onto nitrocellulose, and immunoblotted with specific antibodies. The primary antibodies against phospho-STAT3 (Tyr705), STAT3, phospho-JAK2 (Tyr1007/1008), JAK2, PARP, MCL1, XIAP, Cox2 and iNOS were purchased from Cell Signaling Technology (Danvers, MA). Anti-β-Actin, anti-α-Tublin, anti–mouse IgG and anti–rabbit IgG horseradish peroxidase conjugated antibodies were purchased from Santa Cruz (Santa Cruz, CA). Anti-Myc antibody was purchased from Medical & Biological Laboratories (Tokyo, Japan). Anti-GAPDH antibody was purchased from Abgent (Suzhou, China).

### Cell survival analysis

ESCC cells were seeded in 96-well plates at a density of 5000 cells/100 μl and cultured overnight at 37 °C with 5% CO_2_. After treated with indicated concentrations of curcumin for different times, their survival was measured by CCK-8 assay according to the manufacturer’s protocol as described previously [21]. Absorbance was measured at 450 nm wavelength.

### Colony forming assay

The colony forming assay was performed as described previously [19]. EC9706 cells (2.4 × 10^4^/well) were exposed to various concentrations of curcumin in BME agar with 10% FBS, and then cultured for 2 or 3 weeks at 37 °C in a 5% CO_2_ incubator. Colony numbers were determined by a microscope using Image-Pro Plus software (Media Cybernetics, Inc. Rockville, MD).

### Cell cycle and apoptosis analyses

Cell cycle and apoptosis analyses were performed as described previously [25, 26]. EC9706 (1 × 10^4^/well) were seeded in 96-well plates and incubated overnight, and treated with different doses of curcumin (0, 4, 8, 12, 16 μM) for 24 h. For apoptosis detection, the cells were stained with Hoechst (Beyotime, Shanghai, China) and Annexin V–FITC Kit (Beyotime, Shanghai, China). For cell cycle analysis, the curcumin-treated cells were fixed with 4% paraformaldehyde and stained with DAPI (Solarbio, Beijing, China) according to the protocol and protected from the light for 20 min at 37 °C in a 5% CO2 incubator. They were analyzed by In CELL Analyzer 2000.

### Quantitative real-time polymerase chain reaction (qRT-PCR)

Total RNA was extracted using RNAiso Plus (Takara Bio Group, Japan) according to the manufacturer’s instructions. cDNA was synthesized from equal quantities of total RNA using the PrimeScriptTM RT reagent Kit with gDNA Eraser (Takara Bio Group, Japan). To determine the mRNA levels of Cyclin D1, MCL1 and IL-6, qRT-PCR was performed using SYBR Green qPCR Master Mix (Clontech Laboratories, Inc., USA) with Roche LightCycler® 480II real-time PCR system (Roche, Basel, Switzerland). The primers used were as follows: Cyclin D1, forward 5’-AGCTGTGCATCTACACCGAC-3′ and reverse 5’-GAAATCGTGCGGGGTCATTG-3′; MCL1, forward 5’-GCGACGGCGTAACAAACT-3′ and reverse 5’-ACCCATCCCAGCCTCTTT-3′; IL-6, forward 5’-GTCCAGTTGCCTTCTCCC-3′ and reverse 5’-GCCTCTTTGCTGCTTTCA-3′; GAPDH, forward 5’-GCACCGTCAAGGCTGAGAAC-3′ and reverse 5’-TGGTGAAGACGCCAGTGGA-3′.

### Molecular docking

The *CDOCKER* module of Discovery Studio 3.5 (DS3.5) was used for molecular docking [27]. The crystal structure of JAK2 (PDB entry: 5TQ8) [28] from the RCSB Protein Data Bank (http://www.rcsb.org) was used as the initial structure for molecular docking. The protein structure was prepared with the *Prepare Protein* tool in DS3.5 and curcumin was sketched by using DS3.5 and then prepared with the *Prepare ligands* tool. All parameters were set to default. After preparation of the protein and the compound, curcumin was docked into the active site of JAK2, and all parameters were set to default in the docking process of *CDOCKER*, the protein conformation was fixed and the docked ligand was flexible.

### Protein kinase activity assay

The effect of curcumin on purified kinase JAK2 was assessed using the HotSpot technology kit by Reaction Biology Corp. (Malvern, PA, USA) as described previously [19, 29, 30].

### Patient-derived xenograft (PDX) study

The human ESCC PDX model was established in female CB17/SCID mice (Vital River, Beijing, China). The primary tumors that had passed 3 generations of implantations were inoculated subcutaneously. When the tumors became palpable, the mice were divided into the following four groups (*n* = 8/group): (a) control vehicle (80% DMSO + 20% PBS); (b) curcumin prevention and treatment (Mice were in advance intraperitoneally injected with 100 mg/kg curcumin three times a week for half a month before the formal experiment; 100 mg/kg); (c) curcumin (100 mg/kg); (d) 5-FU(5 mg/kg). Curcumin was intraperitoneal injected three times a week for 4 weeks, and 5-FU was injected continuously for the first 3 days. Body weights and tumors were measured twice a week. Tumors’ volume was calculated as length × width^2^ × 1/2. The study was approved by the Ethics Committee of First Affiliates Hospital of Zhengzhou University and Basic Medical College of Zhengzhou University.

### Immunohistochemistry staining

The paraffin embedding tissue sections were deparaffinized in xylene, treated with a graded series of alcohol and distilled water, and washed thoroughly with TBST. Antigen was retrieval with 0.01 M sodium citrate (pH 6.0) and microwave boiling. Endogenous peroxidase was blocked by using H_2_O_2_. After throughly washing with TBST, the slides were incubated overnight at 4 °C with primary antibodies: p-STAT3, Cox-2 and Caspase-3 diluted in 5% BSA-TBST (1:50) respectively. They were then incubated for 15 min with appropriate dilutions of the secondary antibody at 37 °C for 15 min, and were visualized by incubating with DAB for ~ 5 min. All sections were examined under microscope and analyzed using the HistoQuest 4.0 (TissueGnostics, Austria) as described previously [23].

### TUNEL assay

The paraffin embedding tissue sections were deparaffinized in xylene, treated with a graded series of alcohol and distilled water, and washed thoroughly with PBS. They were then incubated with proteinase K (20 μg/ml in PBS) for 20 min at room temperature, and TUNEL staining was carried out using the in Situ cell death detection kit (KeyGen Biotech Ltd., Nanjing, China) according to the manufacturer’s instructions. TUNEL-positive cells from five independent fields were counted manually.

### Statistical analysis

The results were expressed as mean ± standard deviation (S.D), except otherwise stated. The datas were analyzed by one-way analysis of variance or Student *t* test, using *SPSS* version 17.0 (SPSS, Chicago, USA). *p* < 0.05 was considered statistically significant.

## Results

### Curcumin inhibited the growth of ESCC cells

**Fig. 1:**
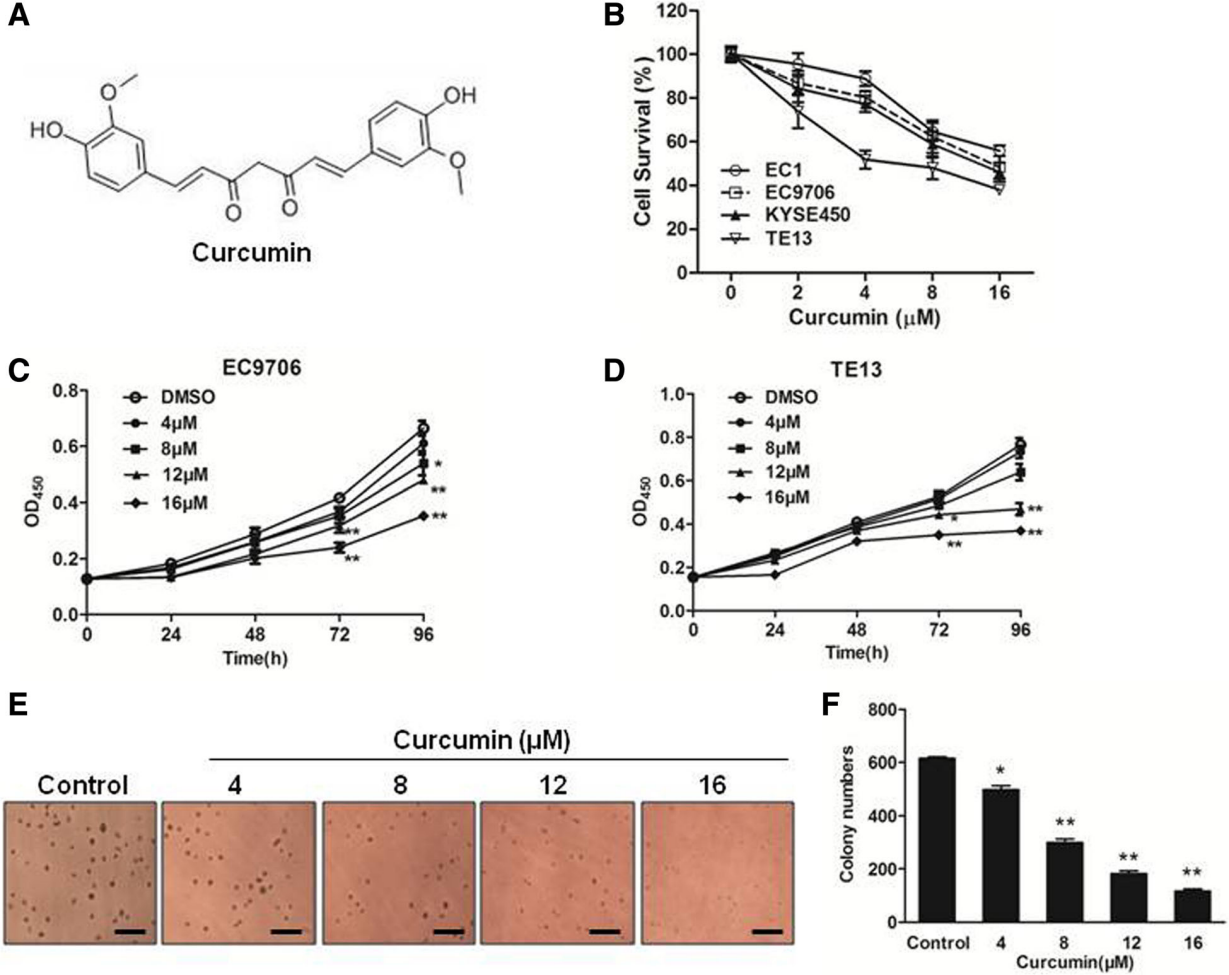
Curcumin inhibited the cell growth of ESCC cells. **a** Chemical structure of curcumin. **b** The cytotoxicity of curcumin in EC1, EC9706, KYSE450 and TE13 cells was evaluated. ESCC cells were treated by indicated concentrations of curcumin for 48 h and then cell viability was measured by CCK-8 assay. **c** and **d** EC9706 cells (**c**) and TE13 cells (**d**) were treated with indicated doses of curcumin at indicated time. Cell viability was measured by CCK-8 assay. **e** and **f** Curcumin reduced colony formation of EC9706 cells in a dose-dependent manner. Soft agar assay was performed as described in materials and methods. A representative picture was taken with 200× magnifications (**e**). Colonies were counted under a microscope and the data was shown as means±SD from triplicate experiments (**f**). ^*^p < 0.05, ^**^p < 0.01

Curcumin is a polyphenol derived from the plant *Curcuma longa* and possess significant anti-cancer activities in various studies. To find whether curcumin could affect esophageal squamous cell carcinoma (ESCC) cell survival, we examined the cytotoxicity of curcumin on four ESCC cell lines. As shown in Fig. 1a and b, curcumin significantly suppressed the survival of the ESCC cells tested dose-dependently. EC9706 and TE13 cells were also treated with indicated concentrations of curcumin for different times. As shown in Fig. 1c and d, curcumin also suppressed EC9706 and TE13 cell growth in a time-dependent manner. Furthermore, we examined the effect of curcumin on anchorage-independent growth of EC9706 cells. Colonies formed in the presence of curcumin were significantly reduced dose-dependently (Fig. 1e and f).

### Curcumin promoted cell cycle arrest and induced cell apoptosis in ESCC cells

**Fig. 2:**
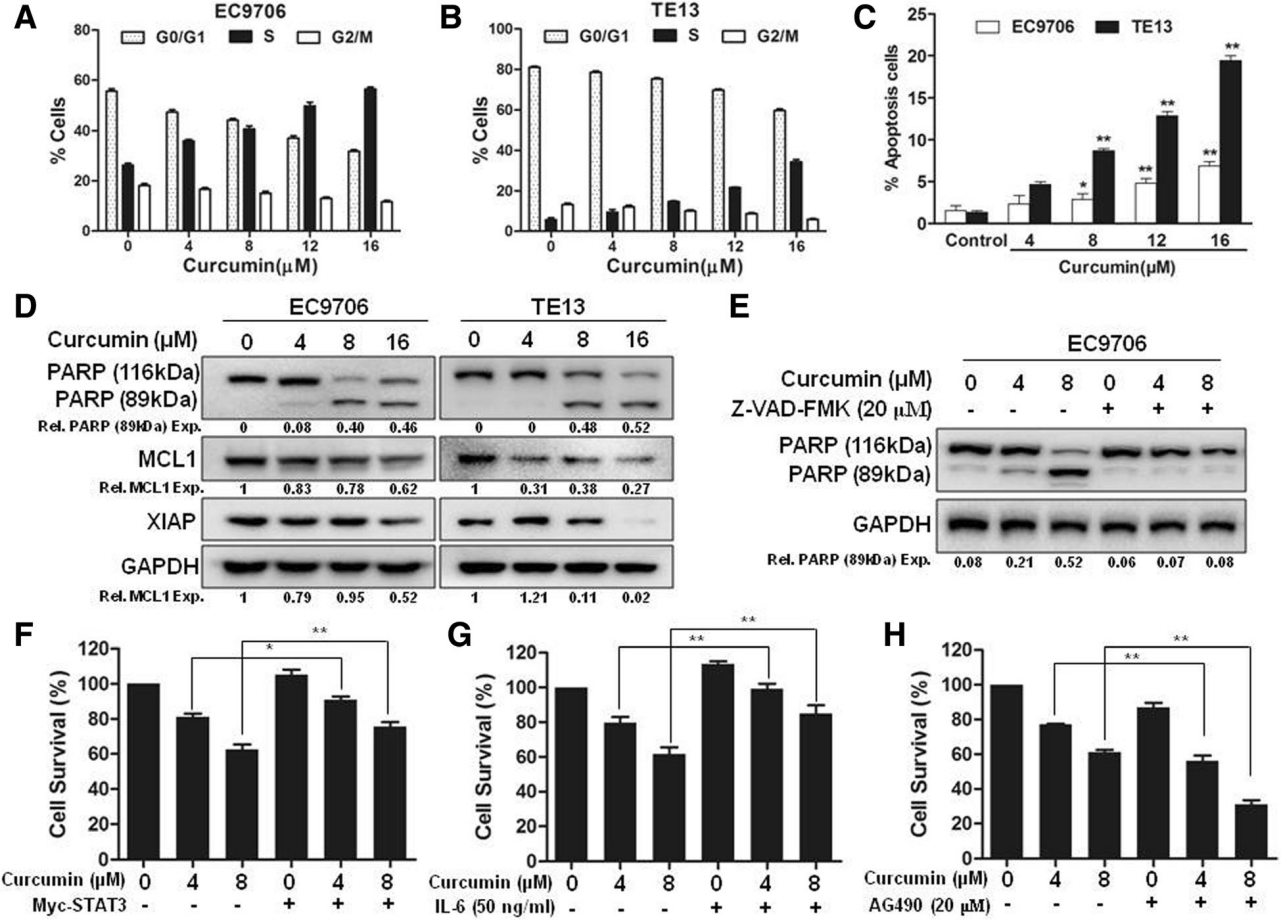
Curcumin promoted cell cycle arrest and induced cell apoptosis in ESCC cells. **a** and **b** EC9706 cells (**a**) and TE13 cells (**b**) were treated with 0, 4, 8, 12 or 16 μM curcumin for 24 h and stained with DAPI. Cell cycle analysis were examined by In CELL Analyzer 2000. Data were shown as means±SD. **c** EC9706 and TE13 cells were treated with indicated concentrations of curcumin for 24 h and then stained with Annexin V-FITC and PI. The percentages of annexin V+ were indicated as means±SD. **d** EC9706 and TE13 cells were treated with 0, 4, 8 or 16 μM curcumin for 24 h and then analyzed by immunoblotting against PARP and anti-apoptotic proteins (MCL1 and XIAP). GAPDH was used as a loading control. **e** EC9706 cells were treated with 0, 4 and 8 μM curcumin or 20 μM Z-VAD-FMK for 24 h and then analyzed by immunoblotting against PARP. GAPDH was used as a loading control. **f** Empty vector (EV) or STAT3 plasmids were transfected into EC9706 cells. Then, the cells were treated with indicated concentrations of curcumin for 24 h, followed by CCK-8 assay. **g** EC9706 cells were treated with 0, 4 and 8 μM curcumin or 50 ng/ml IL-6 for 24 h, and then cells were prepared for CCK-8 assay. **h** EC9706 cells were treated with 0, 4 and 8 μM curcumin or 20 μM AG490 for 24 h, and then cells were prepared for CCK-8 assay. ^*^p < 0.05, ^**^p < 0.01

To further understand the inhibitory action of curcumin on the growth of ESCC cells, we examined the effects of curcumin on cell cycle progression and apoptosis. In the presence of curcumin, there was a significantly increase of EC9706 and TE13 cells arrested at the S phase (Fig. 2a and b). The treatment of EC9706 and TE13 cells with curcumin also led to a markedly increased numbers of apoptotic cells (Fig. 2c). We then evaluated the statuses of caspase substrate PARP in these cells by immunoblotting. As shown in Fig. 2d, curcumin treatment induced the cleavage of PARP dose-dependently in both of EC9706 and TE13 cells. Immunoblotting also showed that the anti-apoptotic proteins MCL1 and XIAP were significantly downregulated by curcumin treatment (Fig. 2d). The curcumin-induced apoptosis was also significantly blocked by Z-VAD-FMK, a potent caspase inhibitor (Fig. 2e). Interestingly, curcumin-induced cell death was significantly attenuated when EC9706 cells were transfected with STAT3-expressing construct (Fig. 2f). Moreover, treatment with IL-6, a STAT3 activating cytokine, substantially inhibited the effect of curcumin on ESCC cell survival (Fig. 2g). In contrast, the presence of AG490, a STAT3 inhibitor, markedly enhanced the effect of curcumin on inducing ESCC cell death (Fig. 2h). These data indicated that curcumin induced apoptosis in ESCC cells, which was affected by STAT3 activation.

### Curcumin inhibited STAT3 activation by suppressing JAK2 phosphorylation in ESCC cells

**Fig. 3:**
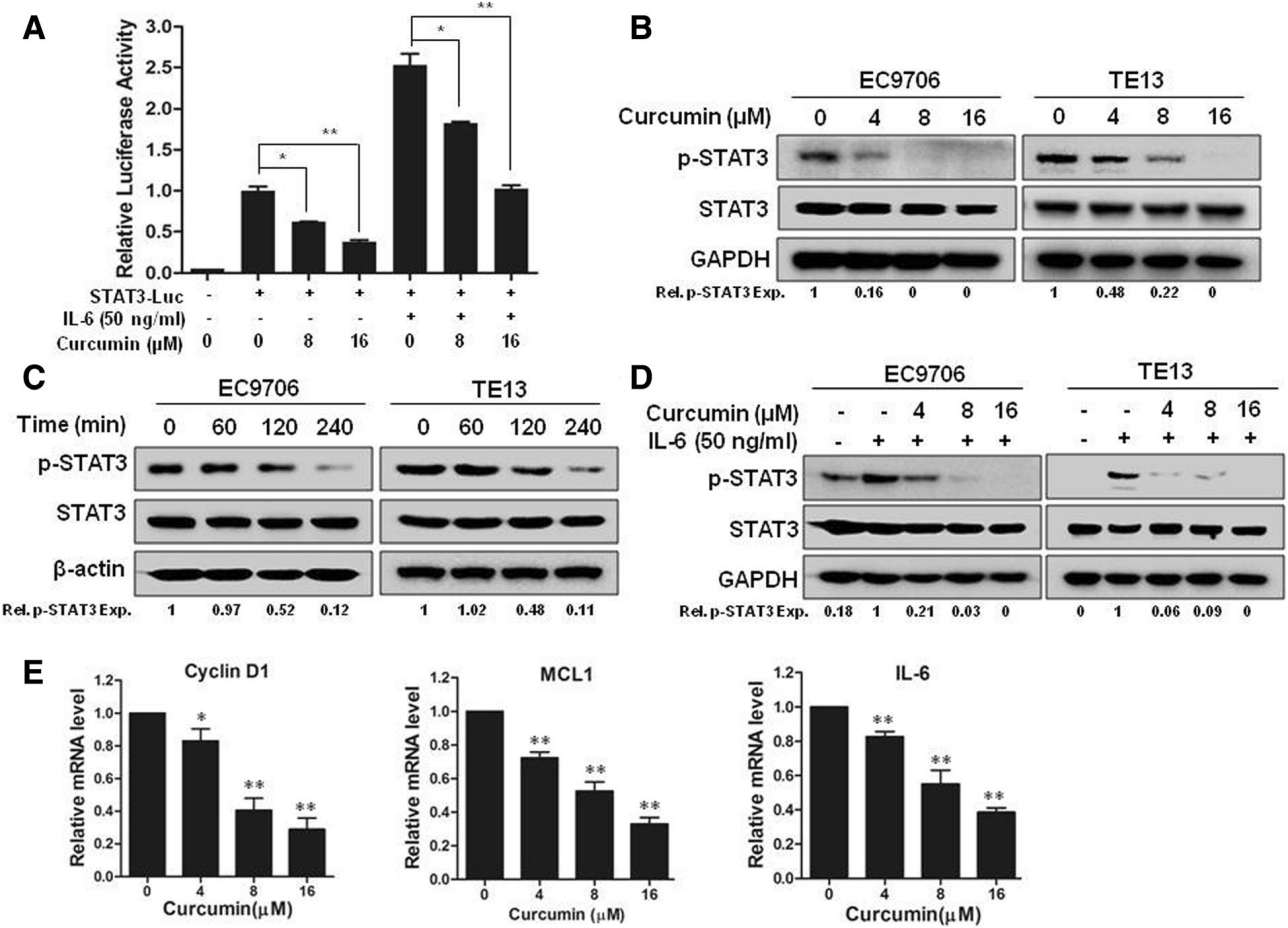
Curcumin inhibited STAT3 activation in ESCC cells. **a** TE13 cells were transfected with vector or STAT3-luc by Lipofectamine^®^ 2000 for 24 h. Cells were then treated with 0, 8 or 16 μM curcumin for 12 h. Luciferase activity was measured followed by stimulation with IL-6 or vehicle control for 20 min. **b** EC9706 and TE13 cells were treated with different concentrations of curcumin for 24 h. Then, cells were prepared for immunoblotting against p-STAT3, STAT3 and GAPDH. **c** EC9706 and TE13 cells were treated with 16 μM curcumin for indicated time, followed by immunoblotting against p-STAT3, STAT3 and β-actin. **d** Following starved overnight in serum-free medium, EC9706 and TE13 cells were incubated with 0, 4, 8 or 16 μM curcumin for 4 h, then stimulated with IL-6 (50 ng/ml) for 20 min. The cells were lysed and analyzed by immunoblotting against p-STAT3, STAT3 and GAPDH. **e** mRNA levels of Cyclin D1, MCL1 and IL-6 were measured by qRT-PCR in TE13 cells treated with 0, 4, 8 or 16 μM curcumin overnight. *p < 0.05, **p < 0.01

The involvement of STAT3 in curcumin-induced apoptosis of ESCC cells propelled us to further examine STAT3 function in these cells. Like the STAT3 inhibitor, curcumin inhibited both spontaneous and IL6-induced STAT3-driven luciferase activities dose-dependently (Fig. 3a). Immunoblotting analysis showed that curcumin decreased the constitutive levels of p-STAT3, but not the total STAT3 in both EC9706 and TE13 cells dose-dependently (Fig. 3b) and time-dependently (Fig. 3c). Moreover, pre-treatment with curcumin blocked IL-6-induced STAT3 phosphorylation in both of EC9706 and TE13 cells (Fig. 3d). To further assess the effect of curcumin on STAT3 activity, the expression levels of several STAT3 target genes were measured by qRT-PCR. As shown in Fig. 3e, the mRNA levels of Cyclin D1, MCL1 and IL-6 were all significantly downregulated by curcumin treatment. Therefore, curcumin effectively suppressed STAT3 phosphorylation and STAT3-mediated transactivation in ESCC cells.

**Fig. 4:**
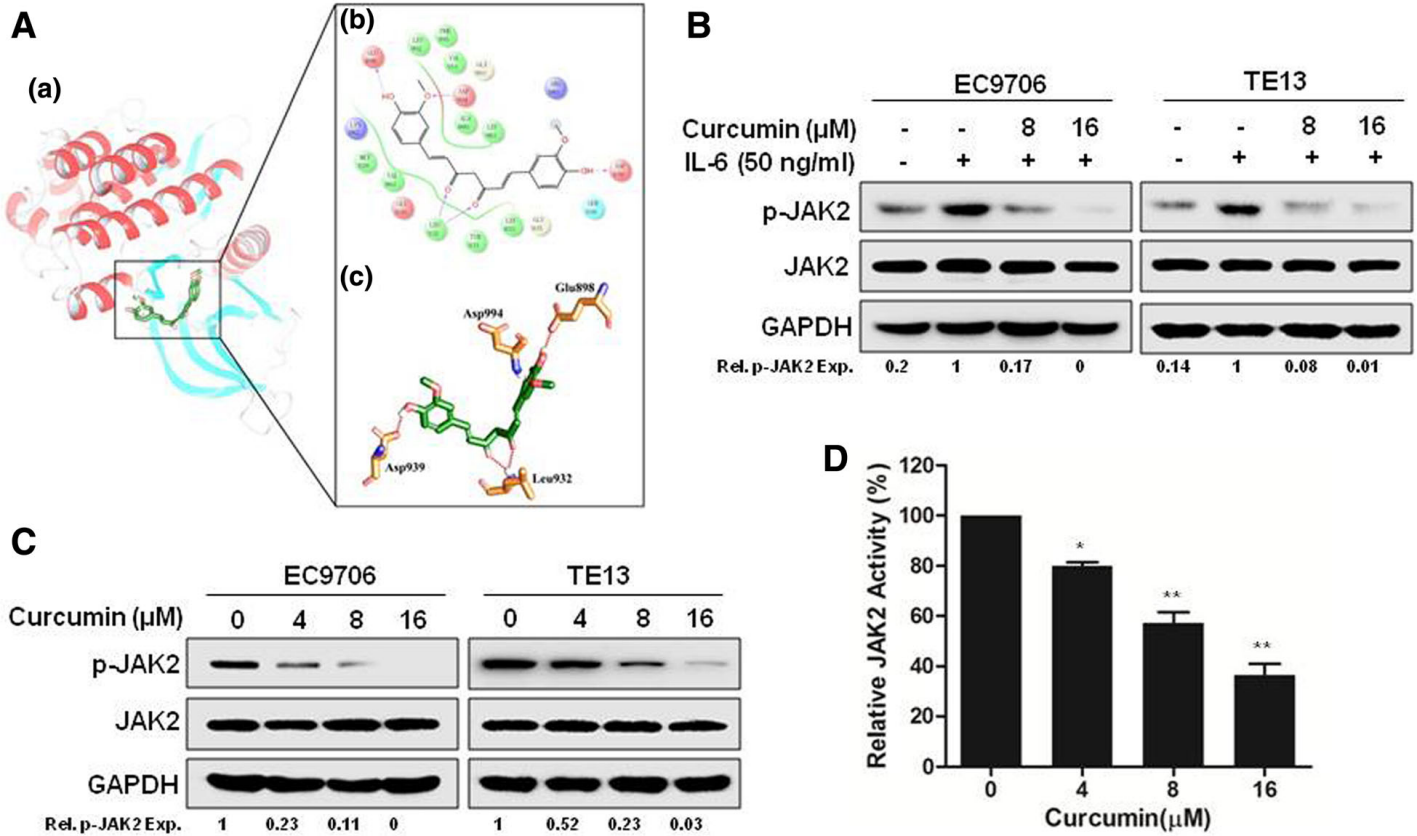
Curcumin inhibits JAK2 phosphorylation in ESCC cells. **a** Molecular modeling of curcumin/JAK2 complex. **a** 3-D presentation of the molecular docking complex pose (curcumin colored in green), **b** 2-D presentation and **c** 3-D presentation of the interactions between curcumin and the key residues of JAK2 (curcumin colored in green, the residues colored in orange, the H-bond colored in red). **b** Following starved overnight in serum-free medium, EC9706 and TE13 cells were incubated with 0, 8 or 16 μM curcumin for 4 h, then stimulated with IL-6 (50 ng/ml) for 20 min. The cells were lysed and analyzed by immunoblotting against p-JAK2, JAK2 and GAPDH. **c** EC9706 and TE13 cells were treated with different concentrations of curcumin for 24 h. Then, cells were prepared for immunoblotting against p-JAK2, JAK2 and GAPDH. **d** JAK2 activity analyses in an in vitro cell-free system. Increasing concentrations of curcumin were incubated with JAK2, and the activity of JAK2 kinase was determined with HotSpot technology. ^*^p < 0.01, ^**^p < 0.001

As JAK2 is an important upstream kinase that phosphorylates STAT3, we analyzed its interaction with curcumin by computer modeling. As shown in Fig. 4a, curcumin was docked nicely into the binding pocket of JAK2. Glu898, Val911, Tyr931, Leu932, Ser936, Asp939 and Asp994 of JAK2 formed strong interactions with curcumin. Among them, Val911, Tyr931 and Ser936 may interact hydrophobically with curcumin, whereas Glu898, Leu932, Asp939 and Asp994 are able to form H-bonds with curcumin. Leu932 in particular can form two H-bonds with curcumin (Fig. 4a). It is conceivable that these H-bonds played a key role in preventing the escape of curcumin from the binding site. We also examined the effect of curcumin on JAK2 experimentally. As shown in Fig. 4b, curcumin markedly inhibited IL6-induced JAK2 phosphorylation in both EC9706 and TE13 cells, similar to its inhibition on STAT3 (Fig. 3d). And the constitutive phosphorylation of JAK2 was also suppressed by curcumin in EC9706 and TE13 cells (Fig. 4c). Furthermore, in the cell-free enzymatic assay, curcumin inhibited JAK2 activity with an IC50 ~ 8 μM (Fig. 4d). Therefore, it is likely that curcumin blocked STAT3 signaling in ESCC cells by inhibiting JAK2 activity.

### Curcumin suppressed tumor growth in ESCC PDX models

**Fig. 5:**
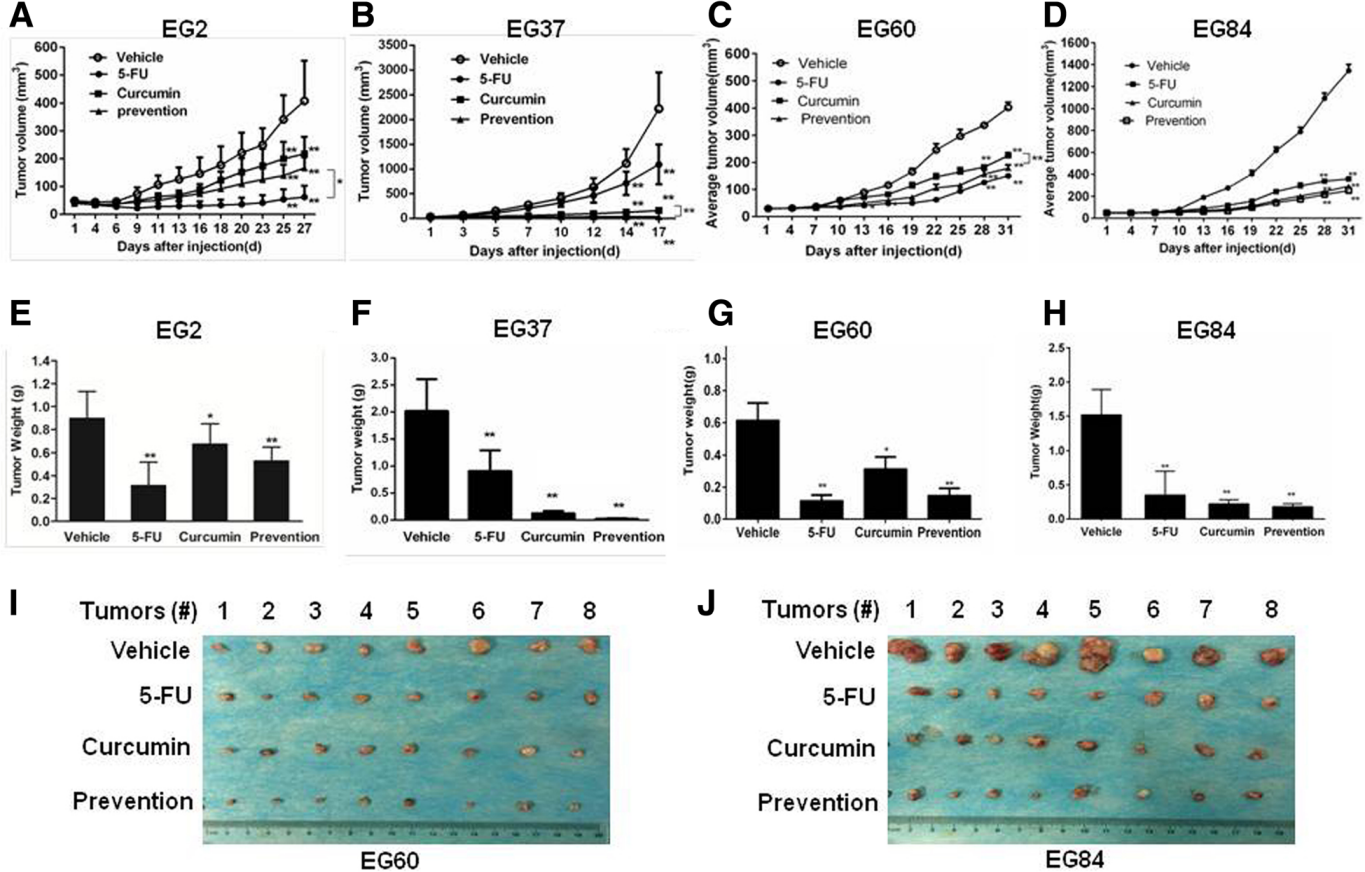
Curcumin suppressed tumor growth in ESCC PDX models. **a-d** When the tumors of the mice injected by EG2 (**a**), EG37 (**b**), EG60 (**c**) or EG84 (**d**) became palpable, the mice were randomly distributed into four groups, and the four groups were administered indicated drugs via intraperitoneal injection three times a week for indicated time. Tumor volumes in each groups were monitored at indicated time. **e-h** Tumors were excised at the end of the experiment, and their weights were measured. **i** and **j** Representative photos of excised tumors in EG60 (I) and EG84 (J) models. ^*^p < 0.05, ^**^p < 0.01

Patient-derived xenograft (PDX) has been proposed as a preferred model to evaluate the effect of targeted therapies for different tumors [31]. To investigate the potential therapeutic effect of curcumin, we established PDXs in SCID mice from primary tumors EG2, EG37, EG60 and EG84. The clinical information of patients EG2, EG37, EG60, and EG84 was summerized in Table 1. Mice with the xenografts were treated with the vehicle, 5-FU, curcumin, or curcumin prevention (mice were in advance intraperitoneally injected with 100 mg/kg curcumin three times a week for half a month before the formal experiment). As shown in Fig. 5a-d, curcumin markedly inhibited the growth of xenografts derived from EG2, EG37, EG60 and EG84. After sacrificed by the end of the treatment, tumor weight from mice received curcumin and curcumin prevention was significantly lower than that from mice given the vehicle (Fig. 5e-h). As a positive control, 5-FU also had notable effect in inhibiting the growth of the xenografts under these experimental conditions. Noteworthily, curcumin prevention group had the most significant inhibition of tumor growth (Fig. 5a-d). The representative photos of the excised tumors from EG60 and EG84 were shown in Fig. 5i and j. These results indicated that curcumin inhibited ESCC tumor growth in the PDX models.

**Table 1:** The clinical pathological features of PDXs

Number	Gender	Age	Tumor Type	TNM Staging	Differentiation
EG2	male	64	oesophagus squamous cell carcinoma	T2N0M0	II
EG37	male	69	oesophagus squamous cell carcinoma	T3N0M0	II
EG60	male	60	oesophagus squamous cell carcinoma	T1N0M0	II
EG84	male	60	oesophagus squamous cell carcinoma	T4N0M0	II

### Curcumin suppressed STAT3 phosphorylation and induced apoptosis in ESCC PDX models

**Fig. 6:**
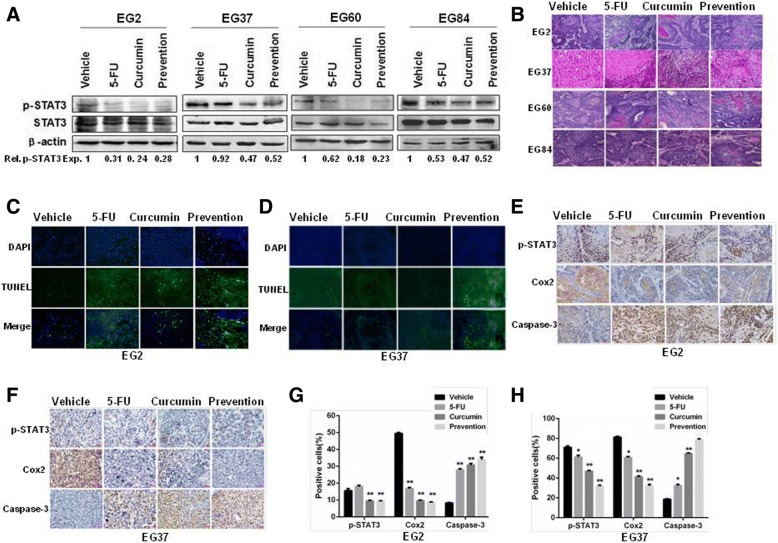
Curcumin suppressed STAT3 phosphorylation and induced apoptosis in ESCC PDX models. **a** The tumor tissues were analyzed by immunoblotting against p-STAT3 and STAT3. β-actin was used as an internal control. **b** Tumor tissues from each group were excised and prepared for HE staining (200×). **c** and **d** Tumor tissues from two PDX models (EG2 and EG37) were prepared for TUNEL assay as described in materials. **e** and **f** Tumor tissues from two PDX models (EG2 and EG37) were prepared for IHC analysis against p-STAT3, Cox2 and Caspase-3. **g** and **h** Statistical analysis of p-STAT3, Cox2 and Caspase-3 from (**e**) and (**f**). ^*^p < 0.05, ^**^p < 0.01

To further evaluate the effect of curcumin on ESCC tumors in PDX models, excised tumor tissues were prepared for immunoblotting analysis. As shown in Fig. 6a, the tumors in animals administrated by curcumin or curcumin prevention contained lower levels of phosphorylated STAT3 than those from vehicle-treated mice. HE staining, TUNEL assay, and IHC analysis were then performed. As shown in Fig. 6b, HE staining indicated that there was less cell growth in tumors from mice received curcumin or curcumin prevention. The TUNEL assay revealed that there were increased apoptotic cells in curcumin-treated tumors (Fig. 6c and d). The IHC analysis with specific antibodies showed that the levels of phosphorylated STAT3 and its target protein Cox2 were markedly decreased in tumors treated with curcumin or curcumin prevention, whereas caspase 3 staining was significantly increased in these tumors (Fig. 6e-h). These data indicated that curcumin or curcumin prevention inhibited PDX tumor growth through inhibiting STAT3 signaling and inducing apoptosis.

## Discussion

STAT3 can be activated by a variety of cytokines including IL-6, IL-10 and TNFα [36]. Constitutive activation of STATs has been found in a number of tumors, including lymphoma, breast cancer, colorectal cancer and prostate cancer [32–35]. Many of the physical, chemical and biological stimuli that induce inflammation, including ultraviolet, reflux gastric juice, and endotoxin, can also activate STAT3 by different mechanisms [37]. Moreover, genes induced by STAT3 can also promote the development of inflammation, forming the positive feedback mechanisms of inflammation development [38]. It has been shown that STAT3 is an important regulator for many genes involved in survival, invasion, cycling, and apoptosis of tumor cells [39, 40]. It has been also demonstrated that down-regulation or inhibition of STAT3 signaling can suppress tumor growth in vitro and in animal models [41]. Thus, STAT3 appears to be a desirable drug target for cancer therapy.

Curcumin is a yellow pigment commonly used as a dietary spice and as a component of tranditional medicine in China and India, primarily for anti-neoplasms and anti-inflammation [42]. Although the compound has not been developed as a drug in modern medicine, it is conceivable that identification of proper biomarkers, such as activated STAT3, might significantly promotes its clinical trials and potential applications in the not-so-distant future. Whasun Lim et al. [43] found that curcumin suppressed proliferation and migration, and induced apoptosis on human placental choriocarcinoma. Gallardo et al. [44] also showed that curcumin inhibited invasive capabilities of breast cancer cells. Our previous study demonstrated that NF-κB signaling pathway was activated in a subgroup of esophageal squamous cell carcinoma (ESCC), and curcumin inhibited ESCC cell proliferation and induced apoptosis [45–47]. Therefore, exploring the effect of curcumin on STAT3 signaling may provides novel therapeutic strategies for ESCC.

In this study, we found that curcumin inhibited the growth of different ESCC cell lines (Fig. 1). It induced cell cycle arrest at S phase and promoted apoptosis dose-dependently in EC9706 and TE13 cells (Fig. 2). Both constitutive and IL6-induced STAT3 activations were strongly inhibited by curcumin treatment in EC9706 and TE13 cells (Fig. 3). Noteworthily, Cox2, which catalyzes the conversion of arachidonic acid to prostaglandins, is inducible by many cytokines in an STAT3-dependent manner [48, 49]. It is highly expressed in many premalignant and malignant lesions, including nasopharyngeal carcinoma, lung cancer, breast cancer [50–52]. A number of studies indicated that Cox2 plays an important role in apoptosis, angiogenesis and tumor invasion [53]. In the present study, we found that the expression of Cox2 was markedly inhibited by curcumin in PDX of ESCC (Fig. 6e-h). We also found that overexpression and activation of STAT3 all decreased the sensitivity of ESCC cells to curcumin (Fig. 2e-g), and the STAT3 inhibition enhanced curcumin-induced ESCC cell death (Fig. 2h). Thus, the effect of curcumin on ESCC cells requires the inhibition of STAT3-mediated signaling.

In the animal experiments, we evaluated the potential therapeutic as well as chemopreventive action of curcumin. Interestingly, as shown in Fig. 5a-c, preventive administration of curcumin was markedly more effective in inhibiting PDXs derived from EG2, EG37 and EG60 than giving curcumin only after innoculation of tumors. In these PDXs, curcumin inhibited STAT3 phosphorylation and the expression of STAT3-regulated Cox2 (Fig. 6e-h). Furthermore, TUNEL assay revealed that apoptosis in the tumor tissues were significantly increased by curcumin treatment (Fig. 6c and d). Taken together, our data indicated that curcumin inhibited JAK2 and blocked the activation of STAT3, leading to decreased expression of STAT3-regulated genes and increased apoptosis in ESCC cells and primary tumors. Therefore, the natural product might be used as an effective chemoprevent agent for ESCC.

## Conclusion

STAT3 signaling is an important mechanism for the development of at least certain ESCC. In this study, we showed that curcumin may inhibit JAK2 and blocked STAT3 signaling pathway, resulting in growth inhibition and apoptosis of these ESCC cells. Therefore, STAT3 system appears to be an effective target in ESCC and curcumin is able to target the system to prevent and treat ESCC.

## Abbreviations

CCK-8: Cell Counting Kit-8
ESCC: Esophageal squamous cell carcinomas
HE staining: Hematoxylin-eosin staining
IHC: Immunohisto-chemistry staining
IL-10: Interleukin-10
IL-6: Interleukin-6
JAK2: Janus kinase 2
PDX: Patient-derived xenograft
qRT-PCR: Quantitative real-time polymerase chain reaction
SDS-PAGE: Sodium dodecylsulfate polyacrylamide gel electrophoresis
STAT3: Signal transducer and activator of transcription 3
TNFα: Tumour necrosis factor alpha
TUNEL: Terminal dexynucleotidyl transferase(TdT)-mediated dUTP nick end labeling
